# OTOF Promotes Clear Cell Renal Cell Carcinoma Progression and Angiogenesis Through AKT-Dependent HIF/VEGFA Signaling

**DOI:** 10.3390/cancers18152498

**Published:** 2026-08-04

**Authors:** Jianhua Wen, Hualin Cao, Jiayin Yu, Jun Huang, Hao Chen, Xinyu Tan, Zhuo Gong, Feng Guo, Zelin Cui, Pengfei Luo

**Affiliations:** 1 Department of Urology, The Central Hospital of Yongzhou, Yongzhou 425006, China; wjhmn@sr.gxmu.edu.cn (J.W.);; 2 Department of Urology, Yongzhou Hospital Affiliated to University of South China, Yongzhou 425006, China; 3 Department of Urology, The First Affiliated Hospital of Guangxi Medical University, Nanning 530007, China; 4 State Key Laboratory of Non-Food Biomass Energy Technology, Institute of Biological Sciences and Technology, Guangxi Academy of Sciences, Nanning 530007, China; 5 Department of Urology, Hunan Provincial People’s Hospital, The First Affiliated Hospital of Hunan Normal University, Changsha 410005, China

**Keywords:** clear cell renal cell carcinoma, angiogenesis, otoferlin, AKT/HIF/VEGFA signaling

## Abstract

Background: Clear cell renal cell carcinoma (ccRCC) is characterized by marked molecular heterogeneity, aggressive clinical behavior, and prominent angiogenesis, highlighting the need to better understand the functional regulators underlying tumor progression and vascular remodeling. OTOF has previously been reported as a prognostically relevant gene in ccRCC; however, its biological function and potential involvement in tumor angiogenesis remain unclear. Methods: In the present study, we investigated the biological and pro-angiogenic roles of OTOF and explored the signaling pathways potentially involved. Results: Analysis of the TCGA-KIRC cohort confirmed that elevated OTOF expression was associated with unfavorable clinical outcomes. Functional experiments demonstrated that OTOF knockdown suppressed ccRCC cell proliferation, migration, and invasion and inhibited xenograft tumor growth. OTOF depletion also reduced intratumoral vascularization and impaired the ability of ccRCC cell-conditioned medium to promote HUVEC tube formation. Mechanistically, OTOF knockdown decreased VEGFA levels and was accompanied by reduced AKT phosphorylation and suppression of downstream HIF/VEGFA signaling. Pharmacological modulation of AKT signaling and VEGFA add-back experiments further supported the functional involvement of the AKT/HIF/VEGFA pathway in OTOF-associated angiogenesis. Conclusions: These findings extend previous observations regarding the prognostic relevance of OTOF by providing functional and mechanistic evidence that OTOF contributes to ccRCC progression and angiogenesis, at least in part, through AKT-dependent HIF/VEGFA signaling.

## Simple Summary

Clear cell renal cell carcinoma is highly vascular and often aggressive. This study examined whether otoferlin (OTOF), a gene associated with poor prognosis, contributes to tumor growth and angiogenesis. Reducing OTOF expression suppressed cancer cell proliferation, migration, invasion, and tumor growth in mice. OTOF knockdown also decreased VEGFA production, tumor vascularization, and the ability of tumor-cell conditioned medium to induce endothelial tube formation. These effects were associated with reduced AKT activation and weaker HIF/VEGFA signaling. Overall, our findings suggest that OTOF promotes ccRCC progression and angiogenesis through an AKT-dependent HIF/VEGFA pathway.

## 1. Introduction

Renal cell carcinoma (RCC) is one of the most common malignancies arising from the renal epithelium and remains a substantial global health burden. Recent epidemiological data indicate that RCC was responsible for 431,288 newly diagnosed cases and 179,368 deaths worldwide in 2020, highlighting its considerable impact on cancer-related morbidity and mortality [1]. Among the histological subtypes, ccRCC is the predominant form, accounting for approximately 75% of all RCC cases [1]. Clinically, surgical resection remains the mainstay of treatment for localized disease; however, a substantial proportion of patients eventually develop recurrence or distant metastasis after initial intervention [2,3]. In the advanced setting, although targeted therapy and immunotherapy have markedly expanded therapeutic options, the clinical management of ccRCC remains challenging because of its pronounced biological heterogeneity, variable therapeutic responsiveness, and the frequent emergence of drug resistance [3]. Moreover, patients with ccRCC generally exhibit less favorable clinical outcomes than those with other histological subtypes of RCC [1]. Therefore, further elucidation of the molecular mechanisms underlying ccRCC progression is essential for the identification of more reliable biomarkers and more effective therapeutic targets [1,3].

ccRCC, the predominant histological subtype of RCC, is characterized by distinctive biological and morphological features, including clear or eosinophilic cytoplasm, abundant intracellular lipid and glycogen accumulation, and a dense, delicate vascular network that reflects its highly angiogenic nature [4,5]. This phenotype is closely linked to dysregulation of the hypoxia signaling pathway, which is a central molecular hallmark of ccRCC. Persistent activation of hypoxia-inducible factor (HIF)-dependent transcription, particularly the HIF2-VEGF axis, drives the expression of multiple pro-angiogenic programs and contributes to the pseudohypoxic state that underlies the characteristic vascularized appearance of this tumor type [5,6]. Given this biological dependency, therapeutic strategies for advanced ccRCC have long centered on inhibition of angiogenic signaling, especially through vascular endothelial growth factor receptor tyrosine kinase inhibitors (VEGFR-TKIs), which have demonstrated meaningful clinical benefit and remain important treatment options in metastatic disease [5,7]. However, despite the initial efficacy of antiangiogenic agents in prolonging disease control, both intrinsic and acquired resistance frequently emerge during treatment, thereby limiting long-term therapeutic success [8,9]. Accumulating evidence indicates that resistance to VEGF-targeted therapies is associated with adaptive molecular reprogramming and activation of alternative oncogenic pathways, ultimately promoting tumor progression and treatment failure [8,9]. Therefore, a more comprehensive understanding of the biological basis of angiogenesis and therapeutic resistance in ccRCC is essential for the development of more selective and durable treatment strategies [5,8,9].

Otoferlin (OTOF) is a large membrane-anchored protein belonging to the ferlin family and contains multiple C2 domains together with a C-terminal transmembrane region. Several of its C2 domains can interact with Ca^2+^, membrane phospholipids, and components of the soluble N-ethylmaleimide-sensitive factor attachment protein receptor (SNARE) machinery. Functional studies in auditory inner hair cells have shown that OTOF acts as a Ca^2+^-responsive regulator of vesicle docking and membrane fusion and is required for efficient synaptic vesicle exocytosis. In addition to exocytosis, OTOF participates in vesicle recycling and clathrin-associated endocytic processes, suggesting that its biological function extends to the coordinated regulation of membrane fusion, retrieval, and vesicle replenishment [10,11,12,13]. These properties indicate that OTOF is not merely a structural membrane protein but a dynamic regulator of Ca^2+^-dependent membrane trafficking.

Although the direct oncogenic functions of OTOF remain poorly characterized, increasing evidence indicates that ferlin-family proteins can influence tumor biology through the regulation of membrane trafficking, receptor stability, intracellular signaling, and secretory activity [11]. In particular, myoferlin has been shown to maintain the membrane abundance and signaling competence of VEGFR2 and to regulate VEGFA secretion in pancreatic cancer cells, thereby promoting tumor-associated angiogenesis [14,15]. These findings provide a family-level mechanistic precedent suggesting that altered ferlin-dependent membrane dynamics may affect both receptor-mediated signaling and the extracellular availability of angiogenic factors. However, these functions have not been directly established for OTOF and therefore remain a hypothesis requiring experimental validation. In ccRCC, previous clinical-expression analyses identified elevated OTOF expression as being associated with unfavorable patient outcomes, but its biological function and mechanistic contribution to tumor progression have not been defined [16]. Given the prominent angiogenic phenotype of ccRCC, we therefore investigated whether OTOF functionally contributes to ccRCC-associated angiogenesis and explored the signaling mechanisms involved [17].

Therefore, in the present study, we investigated the expression, biological function, and pro-angiogenic role of OTOF in ccRCC, with the aim of clarifying its contribution to tumor progression and evaluating its potential as a novel therapeutic target. Through integrated analysis of TCGA data, we found that OTOF was significantly upregulated in ccRCC and was associated with an unfavorable prognosis. Functional experiments further demonstrated that OTOF knockdown markedly inhibited the proliferation, migration, and invasion of renal cancer cells in vitro, and suppressed tumor growth in vivo. In addition, bioinformatics analyses revealed a close association between OTOF and angiogenesis-related programs in ccRCC, which was further supported by both in vitro and in vivo evidence showing that depletion of OTOF attenuated the pro-angiogenic capacity of renal cancer cells. Mechanistically, OTOF promoted ccRCC progression, at least in part, through angiogenesis-related signaling, as silencing OTOF reduced the expression of HIF, VEGFA, and p-AKT. These findings suggest that OTOF may function as a pro-tumorigenic regulator in ccRCC and provide new insight into the molecular basis of ccRCC progression.

## 2. Materials and Methods

### 2.1. Mice and Cell Lines

Male BALB/c nude mice aged 4 weeks were purchased from Sibeifu (Beijing) Biotechnology Co., Ltd. and maintained under specific pathogen-free conditions with controlled temperature, humidity, and a 12 h light/dark cycle. All animal experiments were performed in accordance with institutional guidelines and were approved by the All animal experiments were performed in accordance with institutional guidelines and were approved by IACUC of Top Biotechnology Co., Ltd. (TOPGM-IACUC-2025-0041).

Human ccRCC cell lines, 786-O, Caki-1 and human umbilical vein endothelial cells (HUVECs) were obtained from Procell (CL-0010, CL-0052 and CP-H082Y). 786-O cells were cultured in RPMI-1640 medium supplemented with 10% fetal bovine serum and 1% penicillin–streptomycin (McCOY’s 5A for Caki-1). HUVECs were maintained in Endothelial Cell Medium according to the manufacturer’s instructions (CM-H082Y, Procell). All cells were cultured at 37 °C in a humidified incubator containing 5% CO_2_.

A lentiviral shRNA targeting human OTOF was designed based on a previously validated sequence and synthesized (Tsingke Biotechnology Co., Beijing, China). The OTOF-targeting sequence was 5′-CCTGTCTTTGGGAAGTCCTTT-3′. A non-targeting shRNA was used as the negative control. The shRNA oligonucleotide was cloned into the HIV-1 lentiviral vector, and stable OTOF-knockdown cells were established following antibiotic selection. Knockdown efficiency was confirmed by RT-qPCR and Western blotting.

### 2.2. Tumor Models

To establish the subcutaneous xenograft model, 786-O cells stably expressing the control vector or shOTOF were harvested during the logarithmic growth phase, washed twice with PBS, and resuspended in PBS. A total of 1 × 10^6^ cells in 100 μL of suspension were subcutaneously injected into the flank of each BALB/c nude mouse. A total of 10 mice were randomly allocated to the Vector and shOTOF groups, with five mice per group. Tumor volume was calculated using the following formula: volume = length × width^2^/2. Body weight was recorded at the same time points to monitor the general condition of the animals (the investigators were blinded during tumor measurement). The predefined endpoint was excessive tumor burden (>1000 mm^3^). Animals were euthanized immediately if any humane endpoint was reached. The predefined exclusion criteria included failure of tumor establishment, non-tumor-related illness, accidental injury, or technical failure. For histological quantification, tissue sections and acquired images were assigned coded identifiers and analyzed by an investigator blinded to group allocation.

### 2.3. Human Specimen and Immunohistochemistry, Immunofluorescence

Human ccRCC tissue samples and matched adjacent non-tumor tissues were obtained from three patients who underwent surgical resection at Yongzhou Central Hospital. A total of six tissue specimens were included, comprising three ccRCC tissues and three corresponding adjacent non-tumor tissues. The clinicopathological characteristics of the patients are summarized in Table S1. The histopathological diagnoses were confirmed by experienced pathologists. The collection and use of human tissue samples were approved by the Institutional Ethics Committee of Yongzhou Central Hospital (Approval No.2826051401), and written informed consent was obtained from all patients before tissue collection.

Excised xenograft tumor tissues were fixed in 4% paraformaldehyde, embedded in paraffin, and sectioned at a thickness of 4 μm. For immunohistochemistry, tumor sections were deparaffinized, rehydrated, and subjected to antigen retrieval using citrate buffer, pH 6.0. Endogenous peroxidase activity was blocked with 3% hydrogen peroxide, followed by blocking with BSA. Sections were then incubated overnight at 4 °C with primary antibodies against CD31. After incubation with the appropriate secondary antibody, signals were visualized using a DAB substrate kit and counterstained with hematoxylin. Images were captured using a light microscope, and CD31-positive microvessels were quantified in randomly selected fields.

For immunofluorescence staining, tumor sections were processed similarly for deparaffinization, rehydration, antigen retrieval, and blocking. The sections were incubated with anti-CD31 primary antibody overnight at 4 °C, followed by incubation with fluorescence-conjugated secondary antibody. Nuclei were counterstained with DAPI. Fluorescence images were acquired using a fluorescence or confocal microscope. CD31-positive vascular structures were quantified using ImageJ software or equivalent image analysis software.

### 2.4. Bioinformatics Analysis

Transcriptome expression profiles and corresponding clinical information of patients with KIRC were obtained from The Cancer Genome Atlas KIRC cohort. Transcriptomic data were obtained from the TCGA-KIRC cohort, which primarily represents clear cell renal cell carcinoma. Differentially expressed genes between KIRC tumor tissues and normal kidney tissues were identified using three independent differential expression algorithms, including DESeq2, edgeR, and limma-voom. Genes consistently identified by these methods were considered robust differentially expressed genes for downstream analyses.

Differentially expressed genes were defined using |log2 fold change| > 1 and adjusted *p* value < 0.05. Patients were divided into OTOF-high and OTOF-low groups according to the median OTOF expression level. LASSO Cox regression was performed using the glmnet package, and time-dependent ROC curves were generated using the timeROC package. Gene set enrichment analysis was performed using clusterProfiler, with adjusted *p* value < 0.05 considered statistically significant.

To construct the prognostic signature, survival-related genes were screened and subjected to least absolute shrinkage and selection operator Cox regression analysis. A risk score was calculated for each patient based on the expression levels of selected genes and their corresponding regression coefficients. Patients were stratified into high-risk and low-risk groups according to the median risk score. Kaplan–Meier survival analysis and time-dependent receiver operating characteristic curve analysis were performed to evaluate the prognostic performance of the model. Univariate and multivariate Cox regression analyses were used to assess whether the risk score served as an independent prognostic factor. For mechanistic exploration, KIRC samples were divided into OTOF-high and OTOF-low groups according to OTOF expression. Gene set enrichment analysis was performed to identify biological processes and hallmark pathways associated with OTOF expression. Gene Ontology biological process terms and hallmark gene sets were used as reference gene sets. Enrichment results related to angiogenesis and vascular development were further analyzed and visualized.

The penalty parameter (lambda) of the LASSO-Cox model was determined by 10-fold cross-validation, and lambda.min was used to define the non-zero coefficients (Supplementary Table S1). The robustness of the resulting 10-gene signature was then assessed by internal resampling within the TCGA-KIRC cohort. First, optimism arising from model overfitting was quantified using 1000 bootstrap resamples with the rms package, yielding the apparent and optimism-corrected Harrell concordance index (C-index) and the calibration slope. Second, time-dependent discrimination was evaluated by repeated (20 times) 10-fold cross-validation; in each fold the model was refitted on the training folds and used to compute out-of-fold risk scores for the held-out patients, from which cross-validated 1-, 3-, and 5-year AUCs were calculated with the timeROC package. Third, agreement between predicted and observed survival was examined using bootstrap-based calibration curves at 1, 3, and 5 years. As an additional check, the cohort was randomly partitioned into a training set (70%) and a hold-out validation set (30%); the model was fitted in the training set, and patients in both sets were stratified by the training-derived median risk score for Kaplan–Meier and time-dependent AUC comparison. Analyses were performed in R using the survival, rms, timeROC, and survminer packages.

### 2.5. Cell Proliferation, Invasion, and Migration Assays

Cell proliferation was assessed using the Cell Counting Kit-8 assay. Briefly, Vector and shOTOF cells were seeded into 96-well plates at a density of 5 × 10^3^ cells per well. At the indicated time points, CCK-8 reagent was added to each well and incubated for 2 h at 37 °C. The absorbance at 450 nm was measured using a microplate reader.

For HUVEC viability assays, conditioned media were collected from Vector and shOTOF ccRCC cells and applied to HUVECs. After incubation for the indicated duration, HUVEC viability was detected using the CCK-8 assay as described above.

Cell migration and invasion abilities were evaluated using Transwell chambers. For the migration assay, Vector and shOTOF ccRCC cells suspended in serum-free medium were seeded into the upper chambers, while medium containing 20% FBS was added to the lower chambers as a chemoattractant. For the invasion assay, the upper chambers were pre-coated with Matrigel before cell seeding. After incubation for 48 h, cells remaining on the upper surface of the membrane were removed, whereas migrated or invaded cells on the lower surface were fixed with 4% paraformaldehyde stained with crystal violet, and counted under a microscope in randomly selected fields.

### 2.6. Tube Formation Assay

To evaluate the pro-angiogenic activity of ccRCC cells, conditioned media were prepared from shOTOF 786-O and Caki-1 cells. Briefly, cells were cultured under identical conditions until they reached approximately 70–80% confluence, washed twice with PBS, and incubated in serum-free medium for 24 h. The culture supernatants were collected. Conditioned media were normalized according to the number of viable tumor cells at the time of collection, and equal volumes of normalized conditioned medium were used for all experimental groups. For the tube formation assay, Matrigel was added to pre-cooled 96-well plates and allowed to polymerize at 37 °C for 30 min. HUVECs were resuspended in the indicated conditioned media and seeded onto the Matrigel-coated wells at a density of 3000 cells per well. For VEGFA add-back rescue experiments, recombinant human VEGF-A165 (HEK293, MCE, USA) was added directly to conditioned media from OTOF-knockdown 786-O or Caki-1 cells at a final concentration of 5 ng/mL immediately before HUVEC seeding. An equal volume of the corresponding vehicle was added to the shVector- and shOTOF-conditioned medium control groups. For the positive-control group, HUVECs were cultured in control medium supplemented with recombinant human VEGF-A165 at a final concentration of 25 ng/mL. The experimental groups for each ccRCC cell line included shVector-conditioned medium, shOTOF-conditioned medium, and shOTOF-conditioned medium supplemented with 5 ng/mL VEGF-A165. A separate VEGFA-positive control group containing 25 ng/mL VEGF-A165 was also included. HUVECs were incubated at 37 °C for 6 h, after which representative images were acquired under identical magnification and imaging conditions. Tube formation was quantified using the Angiogenesis Analyzer plugin for ImageJ by measuring the number of branch points and total tube length. The experiments were independently performed four times using independently prepared conditioned media and independently cultured HUVECs. Values from the technical replicates were averaged within each independent experiment, and the quantitative data are presented as the mean ± SD from three independent biological experiments.

### 2.7. Western Blotting

Cells were lysed in RIPA buffer supplemented with protease and phosphatase inhibitors. Total protein concentrations were determined using a BCA protein assay kit. Equal amounts of protein were separated by SDS-PAGE and transferred onto PVDF membranes. After blocking with 5% non-fat milk, membranes were incubated overnight at 4 °C with primary antibodies against OTOF (20721-1-AP, Proteintech, USA, 1:1000), VEGFA (81323-2-RR, Proteintech, USA, 1:25,000), HIF-1α (EPR16897, Abcam, USA, 1:1000), HIF-2α (sc-13596, Santa Cruz, USA, 1:500) AKT (10176-2-AP, Proteintech, USA, 1:7000), phosphorylated AKT (66444-1-Ig, Proteintech, USA, 1:6000), and GAPDH (60004-1-Ig, Proteintech, USA, 1:250000) and ANGPT2 (83816-1-RR, Proteintech, USA, 1:6000). After washing, membranes were incubated with the corresponding horseradish peroxidase-conjugated secondary antibodies (A21010 and A21020, Abbkine, China, 1:50,000). Protein bands were visualized using an enhanced chemiluminescence detection system and quantified using ImageJ software (ver 1.5.0). For detection of VEGFA in conditioned media, equal volumes of conditioned media according to cell number or total protein content were subjected to Western blot analysis. Changes in secreted VEGFA levels were compared between the Vector and shOTOF groups. MK-2206 and SC79(HY-10358, MCE, USA). For pharmacological modulation of AKT signaling, cells were treated with the AKT activator SC79 (HY-18749, MCE, USA) at 10 μM for 1 h to assess acute changes in AKT phosphorylation and for 24 h to evaluate downstream HIF/HIFα and VEGFA expression. For AKT inhibition, cells were treated with MK-2206 (HY-10358, MCE, USA) at 1 μM for 3 h to assess AKT phosphorylation and at 1 μM for 124 h to examine downstream HIF/VEGFA signaling. Original WB images are provided in Supplementary Figures S5–S8.

### 2.8. Quantitative Real-Time PCR

Total RNA was extracted from ccRCC cells using TRIzol reagent (RK30129, Abclonal, China) according to the manufacturer’s instructions. RNA concentration and purity were determined using a spectrophotometer, and samples with an A260/A280 ratio between 1.8 and 2.0 were used for subsequent analyses. A total of 1 μg RNA was reverse-transcribed into cDNA using a reverse-transcription kit (11119ES60, Yeason, China) in accordance with the manufacturer’s protocol. Quantitative real-time PCR was performed using SYBR Green qPCR Master Mix (11201ES03, Yeason, China) on the real-time PCR system. Each reaction was conducted in a total volume of 20 μL. The amplification conditions were as follows: initial denaturation at 95 °C for 30 s/actual duration, followed by 40 cycles of denaturation at 95 °C for 10 s and annealing/extension at 60 °C for 30 s. A melting-curve analysis was subsequently performed from 65 °C to 95 °C to confirm the specificity of amplification. Primer specificity was verified by the presence of a single peak in the melting curve. Primer amplification efficiencies were evaluated using serially diluted cDNA standard curves and were within the acceptable range of 90–110%, with correlation coefficients greater than 0.99. GAPDH was used as the internal reference gene. Relative gene-expression levels were calculated using the 2^ΔΔCt^ method. Each sample was analyzed in three technical replicates, and the experiments were independently repeated three times. The primer sequences were as follows: OTOF forward, 5′-CACGGCCTTCGTCTGGTTC-3′; OTOF reverse, 5′-ATGTAGCCAGGGAGGCTGTA-3′; β-actin forward, 5′-CCTCGCCTTTGCCGATCC-3′; and β-actin reverse, 5′-CATCACGCCCTGGTGCC-3′. HIF-1α forward, 5′-GCCCGCTTCTCTCTAGTCTC-3′; HIF-1α reverse, 5′-CCCTCCATGGTGAATCGGTC-3′; HIF-2α forward, 5′-CTGTATGGTCAGCTCAGCCC-3′; HIF-2α reverse, 5′-GGCTGTCAGACCCGAAAAGA-3′.

### 2.9. Statistical Analysis

All experiments were performed with at least three independent biological replicates unless otherwise indicated. Data are presented as the mean ± standard deviation. Statistical analyses were performed using GraphPad Prism 10.2.3 and R v4.0.3. Comparisons between two groups were conducted using Student’s *t*-test, whereas comparisons among multiple groups were performed using one-way analysis of variance followed by an appropriate post hoc test. Kaplan–Meier survival curves were compared using the log-rank test. Cox regression analyses were used to evaluate prognostic factors associated with overall survival. A two-sided *p* value < 0.05 was considered statistically significant.

## 3. Results

### 3.1. Overview of the TCGA-KIRC Cohort and Identification of Differentially Expressed Genes

**Figure 1:**
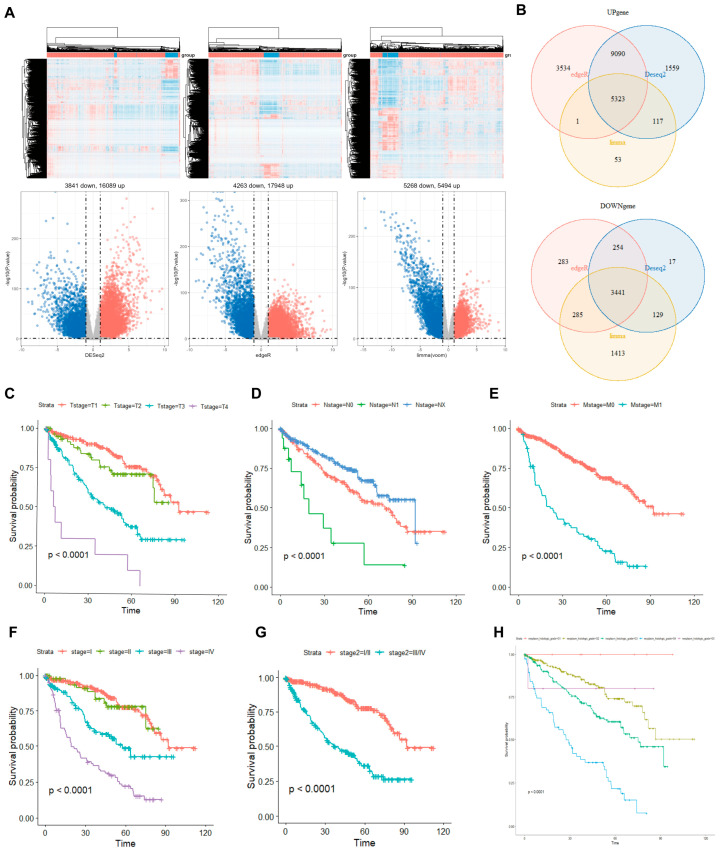
Transcriptomic profiling and clinicopathological survival analysis in KIRC. (**A**) Differentially expressed genes were identified using three independent analytical methods, with representative heatmaps and volcano plots shown for each method. (**B**) Venn diagrams showing the intersecting upregulated and downregulated genes across the three methods. (**C**–**H**) Kaplan–Meier survival analyses of KIRC patients stratified by (**C**) T stage, (**D**) N stage, (**E**) M stage, (**F**) overall stage, (**G**) stage grouping, and (**H**) pathological stage.

To obtain an overview of the transcriptomic landscape of ccRCC, we first analyzed the TCGA-KIRC cohort and visualized the overall distribution of samples and gene expression patterns. Global expression profiling revealed distinct transcriptional differences between tumor and normal kidney tissues. Differential expression analyses were then performed using three independent algorithms, and the intersecting results were used to improve the robustness of candidate gene selection. Through this integrated strategy, a total of 8764 differentially expressed genes (DEGs) were identified in KIRC compared with normal tissues, including 5323 upregulated genes and 3441 downregulated genes (Figure 1A,B and Supplementary Figure S1A). Kaplan–Meier survival analyses were further conducted according to major clinicopathological characteristics in the TCGA-KIRC cohort. The results demonstrated that overall survival was significantly associated with demographic and laboratory variables, including age, hemoglobin level, platelet count, white blood cell count, and serum calcium level. Moreover, tumor-related clinicopathological parameters, including T stage, N stage, M stage, overall stage, tumor grade, and person neoplasm cancer status, were also significantly correlated with prognosis in patients with ccRCC (Figure 1C–H, Supplementary Figure S1B–I). These findings indicate that both clinical features and laboratory indicators are closely linked to survival outcomes in ccRCC and provide an important basis for subsequent identification of prognosis-related genes.

### 3.2. Construction of a 10-Gene Prognostic Signature Identifies OTOF as a Key Prognostic Factor in ccRCC

**Figure 2:**
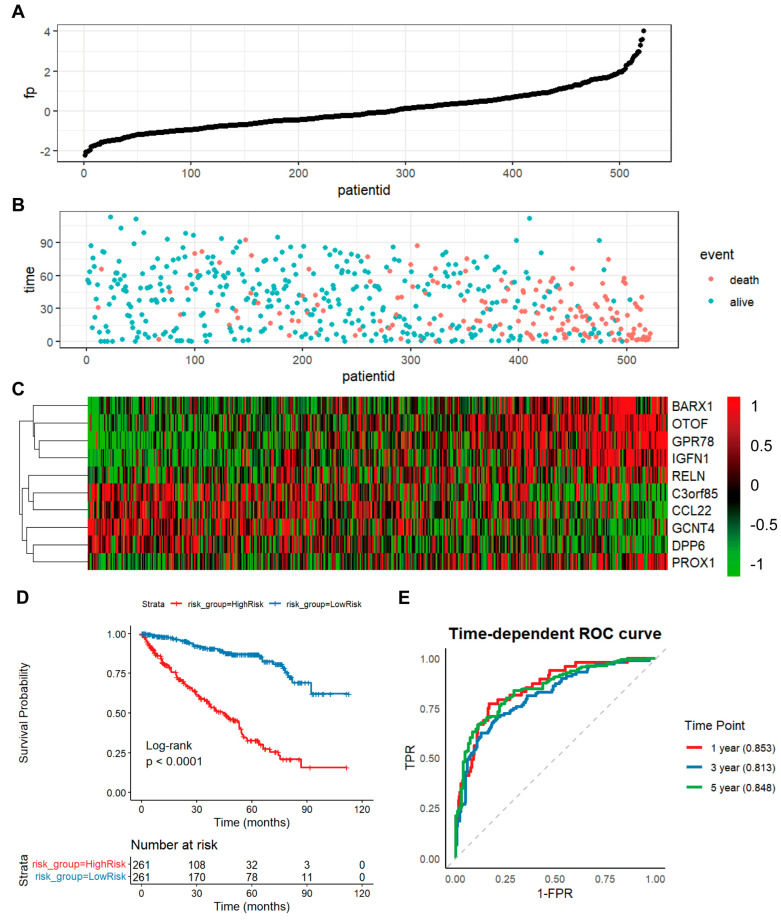
Establishment and evaluation of a prognostic gene signature in KIRC. (**A**–**C**) Distribution of risk scores, survival status, and expression heatmap of the ten signature genes in KIRC patients. (**D**) Kaplan–Meier survival curve comparing overall survival between high- and low-risk groups. (**E**) Time-dependent ROC curves evaluating the predictive performance of the risk model for 1-, 3-, and 5-year survival.

To further identify key genes associated with prognosis in ccRCC, we performed LASSO Cox regression analysis based on gene expression profiles from the TCGA-KIRC cohort. Using this approach, we established a multigene prognostic signature composed of 10 survival-related genes, including OTOF, GPR78, C3orf85, BARX1, DPP6, IGFN1, GCNT4, PROX1, CCL22, and RELN (Figure 2A–C). According to the expression pattern and corresponding regression coefficients of these genes, a risk score model was subsequently constructed for each patient. Based on the median risk score, patients with ccRCC were stratified into high-risk and low-risk groups. Survival analysis demonstrated that patients in the high-risk group exhibited a significantly poorer overall survival than those in the low-risk group (*p* < 0.0001) (Figure 2D). The prognostic performance of this 10-gene signature was further evaluated using time-dependent ROC analysis, which showed that the 1-, 3-, and 5-year AUC values were 0.853, 0.813, and 0.848, respectively, indicating a favorable predictive accuracy of the model (Figure 2E).

To assess whether the apparent performance of the signature was inflated by evaluating the model in the same cohort used for its construction, we performed internal resampling within the TCGA-KIRC cohort. Bootstrap analysis (1000 resamples) showed that the optimism-corrected C-index (0.779) was only marginally lower than the apparent C-index (0.789), corresponding to an optimism of 0.010, and the calibration slope remained close to unity (0.912), indicating minimal overfitting (Supplementary Figure S2A). Consistently, repeated 10-fold cross-validation yielded cross-validated 1-, 3-, and 5-year AUCs of 0.842, 0.796, and 0.831, respectively (Supplementary Figure S2B), which were comparable to the apparent values (0.853, 0.813, and 0.848) and varied little across 20 repetitions (Supplementary Figure S2C). Bootstrap-based calibration curves at 1, 3, and 5 years lay close to the 45-degree reference line, demonstrating good agreement between predicted and observed survival probabilities (Supplementary Figure S2D).

To further illustrate the transferability of the model, we randomly partitioned the cohort into a training set (70%) and a hold-out validation set (30%). Patients stratified by the training-derived median risk score showed clear separation in overall survival in both the training set (*p* < 0.0001) and the independent hold-out set (log-rank *p* = 0.00095) (Supplementary Figure S2E). As expected, the time-dependent AUCs in the hold-out set (0.756, 0.740, and 0.754 at 1, 3, and 5 years) were more conservative than the resampling-based estimates, reflecting the smaller sample size and higher variance of a single split; nevertheless, they still indicated good discrimination in patients not used for model fitting (Supplementary Figure S2F). Taken together, these analyses indicate that the prognostic performance of the 10-gene signature is robust and not substantially overestimated within the TCGA-KIRC cohort (Supplementary Table S2).

**Figure 3:**
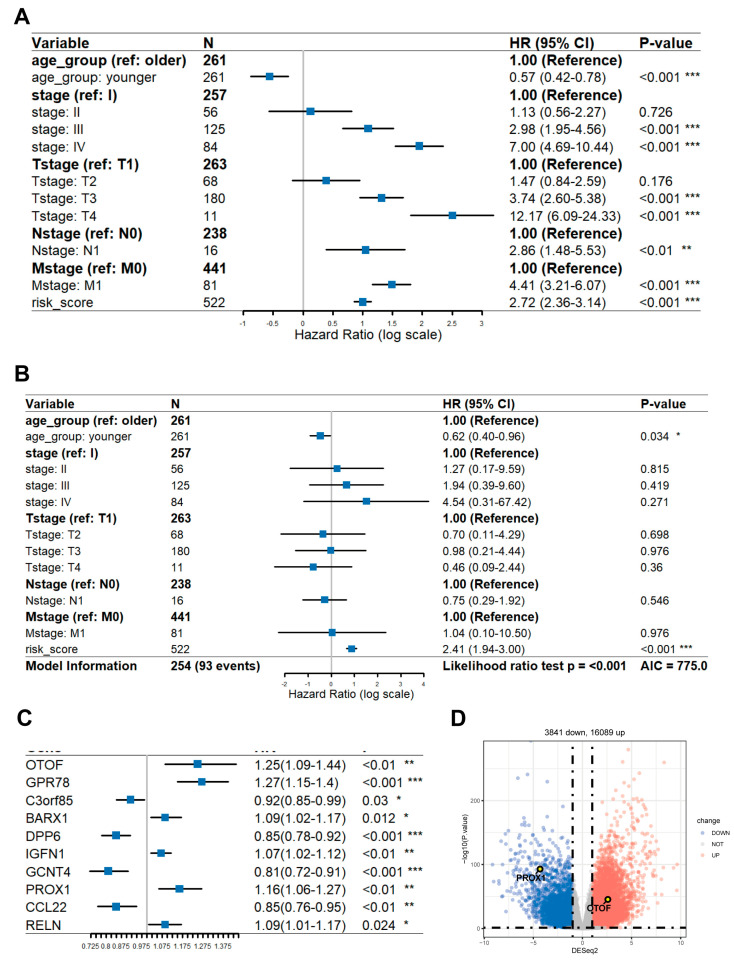
Independent prognostic value of the risk signature in KIRC. (**A**) Univariate Cox regression analysis evaluating the associations between clinicopathological variables, risk score, and overall survival in KIRC patients. (**B**) Multivariate Cox regression analysis confirming the independent prognostic significance of the risk score after adjustment for age, stage, T stage, N stage, and M stage. (**C**) Forest plot showing the prognostic contribution of the ten genes included in the signature. (**D**) Volcano plot highlighting representative candidate genes, including OTOF and PROX1, among differentially expressed genes. * *p* < 0.05, ** *p* < 0.01, *** *p* < 0.001.

To further assess the clinical significance of the risk score, univariate and multivariate Cox regression analyses were performed by integrating the risk score with other important clinicopathological variables. In the univariate analysis, age, stage, T stage, N stage, M stage, and risk score were all significantly associated with overall survival in patients with ccRCC, whereas in the multivariate analysis, risk score remained an independent prognostic factor for patient outcome (Figure 3A,B). These findings suggest that the prognostic signature based on the 10 selected genes has robust predictive value and may serve as an effective tool for risk stratification in ccRCC. In addition, multivariate Cox analysis also revealed distinct contributions of the 10 genes to the risk model. Specifically, OTOF, GPR78, BARX1, IGFN1, PROX1, and RELN were identified as risk-associated genes, whereas C3orf85, DPP6, GCNT4, and CCL22 acted as protective factors, indicating that these genes may play different roles in the occurrence and progression of ccRCC (Figure 3C).

Among these candidate genes, PROX1 and OTOF showed the most remarkable differential expression between tumor and normal tissues. Compared with normal kidney samples, PROX1 was significantly downregulated in ccRCC with a log2FoldChange of −4.31 and an adjusted *p* value of 1.5 × 10^−91^, whereas OTOF was significantly upregulated with a log2FoldChange of 2.58 and an adjusted *p* value of 7.1 × 10^−45^ (Figure 3D). PROX1 showed the most pronounced differential expression among the candidate genes; however, previous studies have already provided functional and mechanistic evidence regarding its role in renal cancer progression. Therefore, further investigation of PROX1 was considered less likely to provide substantial additional biological novelty. In contrast, OTOF showed marked upregulation in ccRCC, a highly significant adjusted *p* value, a strong risk-associated contribution in the prognostic analysis, and a significant association between elevated expression and unfavorable patient survival. Importantly, although OTOF had previously been reported as a prognostically relevant biomarker in ccRCC, its biological function and molecular mechanism in renal cancer had not been experimentally characterized. OTOF was therefore prioritized because it combined strong clinical and prognostic relevance with a substantial unresolved functional and mechanistic gap.

To further address the clinical relevance of OTOF, we performed analyses using the TCGA-KIRC dataset. OTOF mRNA expression was analyzed according to pathological T stage and histological grade and its association with overall survival. These additional results showed that higher OTOF expression was associated with advanced T stage, higher tumor grade, and poorer overall survival (Supplementary Figure S3A–L and Supplementary Table S1).

### 3.3. OTOF Promotes the Malignant Phenotypes of Renal Cancer Cells In Vitro and Tumor Growth In Vivo

**Figure 4:**
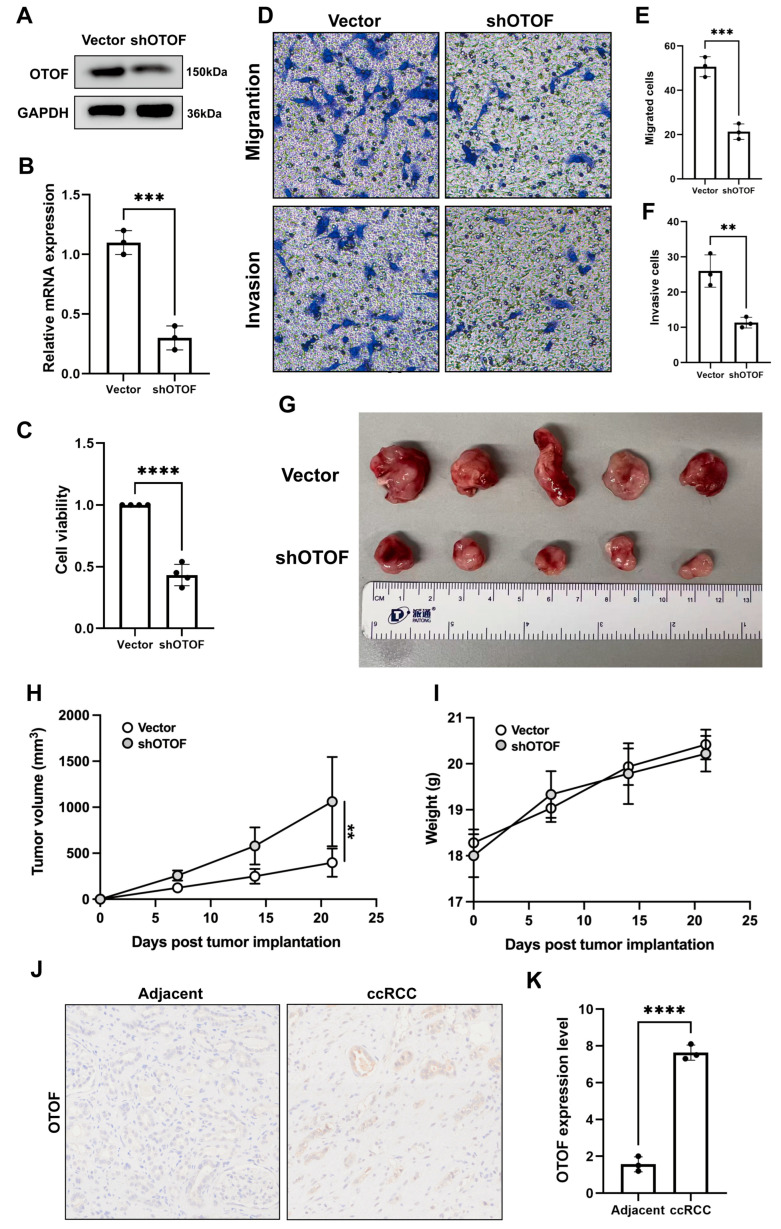
OTOF knockdown suppresses malignant phenotypes and tumor growth of renal cancer cells. (**A**,**B**) Western blotting and qPCR validation of OTOF knockdown efficiency. (**C**) CCK-8 assay showing reduced cell viability after OTOF knockdown in 786-O cells. (**D**–**F**) Representative images and quantification of Transwell migration and invasion assays in Vector and shOTOF cells. (**G**) Representative images of xenograft tumors derived from Vector and shOTOF cells. (**H**) Tumor growth curves showing reduced xenograft tumor volume in the shOTOF group. (**I**) Mouse body weight curves during the xenograft experiment. (**J**,**K**) Representative immunohistochemical staining images of OTOF in human ccRCC tissues and adjacent non-tumorous renal tissues. ** *p* < 0.01, *** *p* < 0.001, and **** *p* < 0.0001.

To further investigate the biological role of OTOF in renal cancer, we first established a stable OTOF-knockdown 786-O cell line (shOTOF). The knockdown efficiency was validated by both Western blotting and qPCR, which consistently showed that OTOF expression in shOTOF cells was markedly lower than that in the vector control group (Figure 4A,B). These results confirmed the successful construction of the OTOF-silenced renal cancer cell model for subsequent functional analyses. We next assessed the effect of OTOF on renal cancer cell viability using the CCK-8 assay. The results showed that cell viability was significantly reduced in the shOTOF group compared with the control group, indicating that OTOF depletion impaired the viability of renal cancer cells (Figure 4C). To further evaluate the effect of OTOF on the malignant behavior of renal cancer cells, Transwell migration and invasion assays were performed. As shown in the representative images and quantitative analyses, the numbers of migrated and invaded cells were both significantly decreased after OTOF knockdown, suggesting that OTOF plays an important role in promoting the migratory and invasive capacities of renal cancer cells (Figure 4D–F). To verify the tumor-promoting effect of OTOF in vivo, a subcutaneous xenograft model was established in nude mice using control and shOTOF 786-O cells (Figure 4G). Tumor growth was monitored over time, and the results demonstrated that xenografts derived from shOTOF cells grew much more slowly than those in the control group. Consistently, the excised tumors in the shOTOF group were markedly smaller than those in the vector group, further confirming that OTOF knockdown significantly suppressed renal cancer growth in vivo (Figure 4H). Notably, there was no obvious difference in body weight between the two groups during the experimental period, suggesting that OTOF silencing inhibited tumor progression without causing overt systemic toxicity (Figure 4I). Immunohistochemical analysis was performed to evaluate OTOF expression in human ccRCC tissues and adjacent non-tumorous renal tissues (n = 3). Representative staining images showed weak or nearly negative OTOF expression in adjacent renal tissues, whereas ccRCC tissues exhibited markedly enhanced OTOF staining (Figure 4J,K). Collectively, these findings indicate that OTOF promotes the malignant phenotypes of renal cancer cells in vitro and facilitates tumor growth in vivo.

### 3.4. OTOF Is Associated with Angiogenesis-Related Programs and Promotes Tumor Vascularization in ccRCC

**Figure 5:**
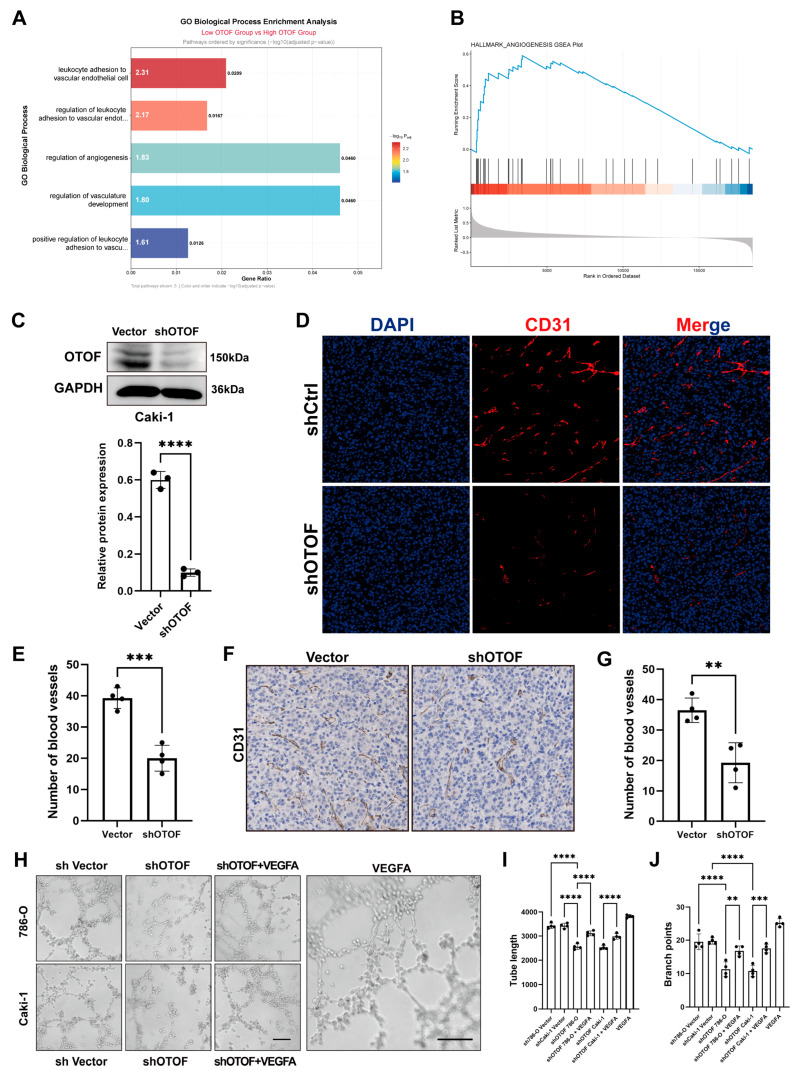
OTOF promotes angiogenesis-associated programs and vascular formation in ccRCC. (**A**) GO biological process enrichment analysis showing enrichment of angiogenesis- and vascular development-related pathways. (**B**) GSEA plot showing enrichment of the HALLMARK_ANGIOGENESIS gene set in the OTOF-high group. (**C**) Western blot analysis confirming the knockdown efficiency of OTOF in Caki-1. (**D**,**E**) Representative CD31 immunofluorescence images and quantification showing reduced tumor vascularization after OTOF knockdown. (**F**,**G**) Representative CD31 immunohistochemistry images and quantification of blood vessel density in xenograft tumors. (**H**) Representative images of HUVEC tube formation following treatment with conditioned media from shVector or shOTOF 786-O and Caki-1 cells, with or without recombinant VEGFA supplementation. (**I**,**J**) Quantification of branch points and total tube length. ns: not significant (*p* > 0.05), ** *p* < 0.01, *** *p* < 0.001, and **** *p* < 0.0001.

Given the pronounced effect of OTOF on ccRCC progression, we next sought to investigate the potential mechanism by which OTOF contributes to malignant tumor development. To this end, ccRCC samples from the TCGA cohort were stratified into OTOF-high and OTOF-low groups according to OTOF expression levels, followed by gene set enrichment analysis. GO biological process analysis revealed that OTOF-associated genes were significantly enriched in angiogenesis- and vascular development-related biological processes, including regulation of angiogenesis, regulation of vasculature development, leukocyte adhesion to vascular endothelial cells, and regulation of leukocyte adhesion to vascular endothelial cells (Figure 5A). Consistently, HALLMARK GSEA further demonstrated significant enrichment of the HALLMARK_ANGIOGENESIS gene set in the OTOF-high group (Figure 5B). These findings suggested that OTOF may participate in ccRCC progression by modulating angiogenesis-related biological programs.

To experimentally validate the association between OTOF and tumor angiogenesis, we next examined vascular formation in xenograft tumor tissues derived from the aforementioned in vivo experiments. Immunohistochemical staining for CD31, a canonical endothelial marker, showed that CD31-positive vascular structures were markedly reduced in tumors from the shOTOF group compared with those from the Vector control group. Quantitative analysis further confirmed a significant decrease in microvessel density following OTOF knockdown (Figure 5F,G). In parallel, immunofluorescence staining of CD31 revealed abundant and well-organized vascular structures in control tumors, whereas OTOF-depleted tumors displayed substantially weaker CD31 signals and fewer vascular structures. Consistent with the immunohistochemical results, quantification of CD31-positive vessels demonstrated that OTOF knockdown significantly impaired tumor vascularization in vivo (Figure 5D,E). These results indicate that OTOF depletion suppresses angiogenesis within ccRCC xenograft tumors.

We further investigated whether OTOF regulates the pro-angiogenic capacity of ccRCC cells in vitro. Conditioned media were collected from shVector control and shOTOF 786-O and Caki-1 cells, with OTOF knockdown in Caki-1 cells validated in Figure 5C, and then applied to HUVECs to assess endothelial tube formation. Compared with the corresponding shVector-conditioned media, conditioned media derived from OTOF-knockdown cells markedly attenuated the ability of HUVECs to form capillary-like networks. Quantitative analysis showed that both the number of branch points and the total tube length were significantly reduced in the shOTOF-conditioned medium groups (Figure 5H–J). To determine whether the impaired angiogenic activity caused by OTOF depletion was mediated, at least in part, by reduced VEGFA availability, recombinant VEGFA was supplemented into conditioned media derived from OTOF-knockdown 786-O and Caki-1 cells. VEGFA supplementation partially restored HUVEC tube formation, as reflected by increased branch point formation and total tube length compared with the corresponding shOTOF-conditioned medium groups. These findings indicate that reduced VEGFA availability contributes substantially to the impaired pro-angiogenic activity caused by OTOF depletion and suggest that OTOF enhances the paracrine pro-angiogenic capacity of ccRCC cells, at least in part, through VEGFA-dependent signaling.

Collectively, these bioinformatic and experimental results demonstrate that OTOF is closely associated with angiogenesis-related pathways and functionally promotes tumor angiogenesis in ccRCC. OTOF knockdown significantly reduced vascular formation in xenograft tumors and impaired the ability of ccRCC cell-derived conditioned medium to induce endothelial tube formation, supporting a critical role for OTOF in enhancing the angiogenic potential of ccRCC.

### 3.5. OTOF Promotes VEGFA-Mediated Angiogenesis Through Activation of the AKT/HIF Signaling Axis in ccRCC

**Figure 6:**
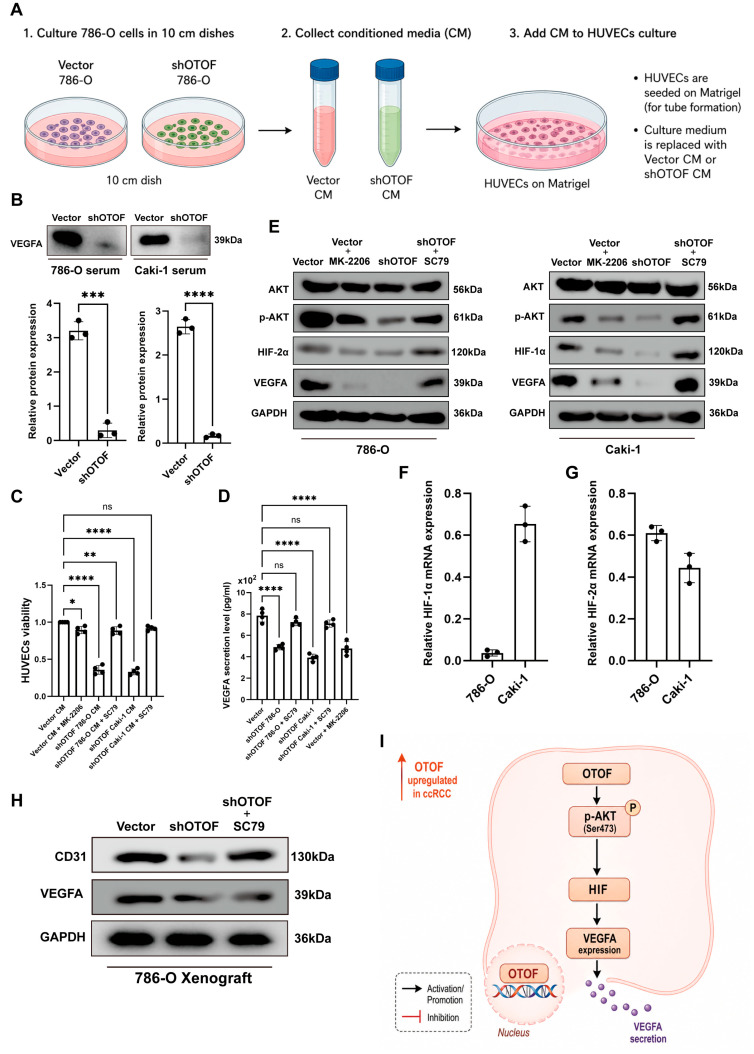
OTOF knockdown attenuates angiogenesis-related phenotypes and AKT/HIF/VEGFA signaling in ccRCC. (**A**,**B**) Schematic illustration and Western blot analysis showing VEGFA expression in conditioned medium derived from Vector and shOTOF 786-O cells. (**C**) ELISA quantification of VEGFA secretion levels in conditioned media from Vector and shOTOF ccRCC cells. (**D**) HUVEC viability assay showing that conditioned media from OTOF-knockdown 786-O and Caki-1 cells significantly suppressed endothelial cell viability. (**E**) Western blot analysis of AKT, p-AKT, HIF (Caki-1: HIF-1α; 786-O: HIF-2α) and VEGFA expression in 786-O and Caki-1 cells treated with Vector, Vector plus the AKT inhibitor MK-2206, shOTOF, or shOTOF plus the AKT activator SC79. (**F**) qPCR analysis of relative mRNA expression of HIF-1α in 786-O and Caki-1 cells, respectively. (**G**) qPCR analysis of relative mRNA expression of HIF-2α in 786-O and Caki-1 cells, respectively. (**H**) Western blot analysis of angiogenesis-related proteins in 786-O xenograft tumors. OTOF knockdown decreased the expression of CD31 and VEGFA, whereas SC79 treatment partially rescued their expression. (**I**) Proposed mechanism by which OTOF promotes angiogenesis in ccRCC through activation of the AKT/HIF/VEGFA signaling axis. ns: not significant (*p* > 0.05), * *p* < 0.05, ** *p* < 0.01, *** *p* < 0.001, and **** *p* < 0.0001.

Based on these findings, we hypothesized that OTOF may promote angiogenesis by modulating the paracrine secretion of pro-angiogenic factors from ccRCC cells. Since VEGFA is one of the most potent and well-characterized mediators of tumor angiogenesis, we first examined VEGFA levels in conditioned medium derived from Vector and shOTOF ccRCC cells. Western blot analysis showed that VEGFA abundance was markedly reduced in the conditioned medium from shOTOF cells compared with that from Vector cells (Figure 6A,B). Consistently, ELISA further confirmed a significant decrease in secreted VEGFA levels following OTOF knockdown (Figure 6C). In addition to its inhibitory effect on endothelial tube formation, conditioned medium from shOTOF cells also significantly reduced the viability of HUVECs, as determined by a CCK-8 assay (Figure 6D), suggesting that OTOF depletion attenuates the paracrine pro-angiogenic activity of ccRCC cells.

To further explore the signaling mechanism underlying OTOF-mediated VEGFA regulation, we examined the AKT/HIF axis, a canonical upstream pathway known to promote VEGFA expression under tumor-associated conditions [18,19,20]. Western blot analysis showed that OTOF knockdown markedly reduced AKT phosphorylation, HIF-1α and HIF-2α expression, and intracellular VEGFA levels in both 786-O and Caki-1 cells, whereas total AKT expression remained largely unchanged. Consistently, pharmacological inhibition of AKT with MK-2206 in vector control cells mimicked the effects of OTOF knockdown, leading to decreased AKT phosphorylation accompanied by reduced HIF-1α (Caki-1), HIF-2α (786-O) and VEGFA expression. In contrast, treatment with the AKT activator SC79 restored AKT phosphorylation and substantially rescued HIF-1α (Caki-1), HIF-2α (786-O) and VEGFA expression in both ccRCC cell lines (Figure 6E). These findings indicate that OTOF promotes VEGFA expression, at least in part, through activation of the AKT-Dependent HIF/VEGFA Signaling.

To further validate the role of this pathway in tumor angiogenesis in vivo, we examined angiogenesis-related proteins in 786-O xenograft tumors. OTOF knockdown markedly reduced the expression of CD31 and VEGFA, indicating impaired tumor vascularization. Notably, SC79 treatment partially restored the expression of these angiogenesis-associated markers in shOTOF xenografts (Figure 6F). Together, these results suggest that OTOF facilitates ccRCC angiogenesis by activating AKT signaling and subsequently enhancing the HIF/VEGFA axis. Pharmacological reactivation of AKT partially reverses the inhibitory effects of OTOF knockdown on VEGFA expression and angiogenesis-related molecular changes, supporting a mechanistic role of AKT signaling in OTOF-mediated angiogenesis in ccRCC (Figure 6G).

## 4. Discussion

Previous studies have reported that OTOF is upregulated in ccRCC and that elevated OTOF expression is associated with unfavorable patient outcomes [18]. Therefore, the prognostic association of OTOF itself does not represent the principal novelty of the present study. Rather, our findings extend the existing clinical observations by providing functional and mechanistic evidence for the biological role of OTOF in ccRCC. We demonstrate that OTOF depletion suppresses malignant cellular phenotypes and xenograft growth and, importantly, reduces the pro-angiogenic activity of ccRCC cells. OTOF knockdown decreased VEGFA production and secretion, impaired HUVEC tube formation, and reduced tumor vascularization. In addition, pharmacological modulation of AKT signaling and VEGFA add-back experiments supported the involvement of AKT-dependent HIF/VEGFA signaling in the angiogenic phenotype associated with OTOF. Thus, the main contribution of this study is the functional characterization of OTOF as a pro-tumorigenic and pro-angiogenic regulator rather than its initial identification as a prognostic marker.

ccRCC is characterized by substantial interpatient heterogeneity, which contributes to marked differences in clinical outcomes even among patients with similar clinicopathological features [19]. Therefore, molecular signatures that capture the biological diversity of ccRCC may provide additional prognostic information beyond conventional staging systems. In this study, we first identified differentially expressed genes in the TCGA-KIRC cohort and constructed a 10-gene prognostic signature with robust risk-stratification capacity. This multigene model may reflect the complex molecular landscape underlying ccRCC progression and provide a useful framework for prognostic evaluation. However, rather than treating the signature solely as a predictive tool, we further focused on genes with potential biological and clinical relevance. Among these candidates, OTOF emerged as a particularly noteworthy factor, as it was incorporated into the prognostic model and its elevated expression was associated with unfavorable patient outcomes. OTOF has been primarily studied in the context of hereditary hearing loss, membrane trafficking, calcium-dependent vesicle exocytosis, and synaptic vesicle release [20,21,22]. In contrast, its role in cancer remains poorly defined, and its contribution to renal cancer progression has not been systematically investigated. These observations suggest that OTOF may represent not only a prognostic indicator but also a previously underappreciated functional regulator in ccRCC. Thus, the identification of OTOF from the prognostic signature provided a strong rationale for subsequent experimental validation and mechanistic exploration.

Importantly, our functional experiments further demonstrated that OTOF is not merely a prognostic marker, but may act as a functional contributor to ccRCC progression. Consistent with the bioinformatic findings, silencing OTOF markedly suppressed the malignant phenotypes of ccRCC cells, including cell proliferation, migration and invasion, suggesting that OTOF is required for maintaining the aggressive behavior of renal cancer cells. These in vitro observations were further supported by the xenograft model, in which OTOF knockdown significantly restrained tumor growth in vivo. Thus, the prognostic implication of OTOF was experimentally validated at the functional level, strengthening the biological relevance of OTOF in ccRCC. Given that tumor progression is driven not only by intrinsic proliferative capacity but also by enhanced motility, invasiveness and adaptation to the tumor microenvironment [23,24,25,26], our findings indicate that OTOF may participate in multiple steps of ccRCC progression. Collectively, these results identify OTOF as a potential oncogenic regulator in ccRCC and provide a functional basis for further investigating the molecular mechanisms by which OTOF promotes renal cancer development.

Angiogenesis is a defining biological feature of ccRCC and plays a central role in tumor growth, metastatic dissemination, and therapeutic response [27,28,29]. Owing to frequent dysregulation of hypoxia-related signaling and vascular regulatory programs, ccRCC is generally regarded as one of the most highly vascularized solid tumors [30]. Consistent with this biological context, our enrichment analyses revealed that OTOF-associated genes were significantly involved in angiogenesis-related processes, suggesting a potential link between OTOF and vascular remodeling in ccRCC. This prediction was further supported by our experimental findings. CD31 staining of xenograft tumors showed that OTOF knockdown markedly reduced intratumoral microvessel formation, while conditioned medium from OTOF-silenced ccRCC cells significantly impaired HUVEC viability and tube formation in vitro. These observations indicate that OTOF may enhance the pro-angiogenic capacity of ccRCC cells, at least in part, through paracrine regulation of endothelial cells. Mechanistically, VEGFA is one of the most potent and well-established tumor-derived pro-angiogenic factors, and its expression is tightly controlled by upstream oncogenic and hypoxia-responsive signaling pathways, including the AKT/HIF axis. In our study, OTOF knockdown decreased VEGFA levels both in ccRCC cells and in the conditioned medium, as confirmed by Western blotting and ELISA. Moreover, silencing OTOF reduced the expression of HIF and phosphorylated AKT, whereas total AKT expression remained largely unchanged. These findings suggest that OTOF may primarily affect AKT activation rather than total AKT abundance, thereby attenuating HIF-mediated VEGFA production and weakening the angiogenic crosstalk between ccRCC cells and endothelial cells. Collectively, these results identify the AKT/HIF/VEGFA signaling axis as a potential downstream mechanism through which OTOF promotes angiogenesis and contributes to ccRCC progression.

The precise molecular mechanism by which OTOF influences AKT activation remains to be elucidated. OTOF is a multi C2 domain protein with established roles in Ca^2+^-dependent membrane trafficking and membrane fusion [12]. Therefore, rather than directly interacting with AKT, OTOF may influence AKT phosphorylation indirectly by modulating the trafficking, membrane localization, recycling, or stability of upstream receptors and signaling complexes that converge on the PI3K/AKT pathway. This possibility is conceptually supported by studies of other ferlin family members, such as myoferlin, which can regulate the membrane abundance and signaling activity of receptor tyrosine kinases [14,31]. Alternatively, OTOF-dependent alterations in Ca^2+^-regulated membrane trafficking or secretory activity may modify autocrine or paracrine signaling inputs that subsequently affect AKT activation. However, these possibilities remain speculative and require direct experimental validation. Thus, our present findings support the functional involvement of AKT signaling downstream of OTOF but do not establish that OTOF directly activates AKT.

Despite these findings, several limitations should be acknowledged. First, our functional experiments were primarily based on OTOF knockdown models; therefore, complementary OTOF overexpression assays would further strengthen the causal relationship between OTOF expression and ccRCC progression. Second, although our data suggest that OTOF knockdown suppresses AKT phosphorylation, HIF expression, and VEGFA production, the precise molecular mechanism by which OTOF regulates AKT activation remains to be elucidated. Third, although the major in vitro angiogenesis-related and signaling findings were validated in two independent ccRCC cell lines, 786-O and Caki-1, the in vivo xenograft experiments were performed using only the 786-O model. Given the substantial molecular heterogeneity of ccRCC, additional validation in other cell lines would further strengthen the generalizability of these findings. In addition, because the prognostic signature was mainly constructed and evaluated using the TCGA-KIRC cohort, further validation in independent external cohorts is required before clinical application.

## 5. Conclusions

Collectively, our findings identify OTOF as a previously unrecognized prognostic and pro-angiogenic regulator in ccRCC. Integrative transcriptomic and survival analyses revealed the clinical relevance of OTOF in renal cancer progression, while in vitro and in vivo experiments demonstrated that OTOF knockdown suppresses tumor cell viability, migration, invasion, xenograft growth, and angiogenic potential. Mechanistically, OTOF depletion impaired AKT phosphorylation and reduced HIF and VEGFA expression, whereas pharmacological activation of AKT by SC79 partially restored the HIF/VEGFA axis. These results suggest that OTOF promotes ccRCC progression and angiogenesis, at least in part, through activation of the AKT/HIF/VEGFA signaling pathway. Therefore, OTOF may represent a potential therapeutic research direction requiring further validation.

## Data Availability

The publicly available transcriptomic and clinical data analyzed in this study were obtained from The Cancer Genome Atlas Kidney Renal Clear Cell Carcinoma cohort (TCGA-KIRC) through the National Cancer Institute Genomic Data Commons Data Portal. The project identifier is TCGA-KIRC. The experimental data generated in this study are included in the article and its Supplementary Materials. Additional raw experimental data are available from the corresponding author upon reasonable request.

## Supplementary Materials

The following supporting information can be downloaded at: https://www.mdpi.com/article/10.3390/cancers18152498/s1, Figure S1: Transcriptomic characteristics and clinical stratification analyses of KIRC patients; Figure S2: Internal validation and performance evaluation of the OTOF-based prognostic signature; Figure S3. Prognostic and clinical significance of candidate genes and OTOF in KIRC patients; Figure S4. Quantification of Western blot analyses showing OTOF-associated regulation of AKT/HIF/VEGFA signaling and angiogenesis-related proteins. Figure S5. Original WB images of Figure 4A, Figure 5C and Figure 6B; Figure S6. Original WB images of Figure 6E in 786-O; Figure S7. Original WB images of Figure 6E in Caki-1; Figure S8. Original WB images of Figure 6H; Table S1: The key characteristics; Table S2: Internal validation results.
