# Supplementary material for: OTOF Promotes Clear Cell Renal Cell Carcinoma Progression and Angiogenesis Through AKT-Dependent HIF/VEGFA Signaling
Source: Cancers (Basel). 2026 Aug 4;18(15):2498. doi: 10.3390/cancers18152498 (PMC13463935; doi:10.3390/cancers18152498)
Supplement: Supplementary file 1 [file cancers-18-02498-s001.zip › cancers-4417606-supplementary.pdf]

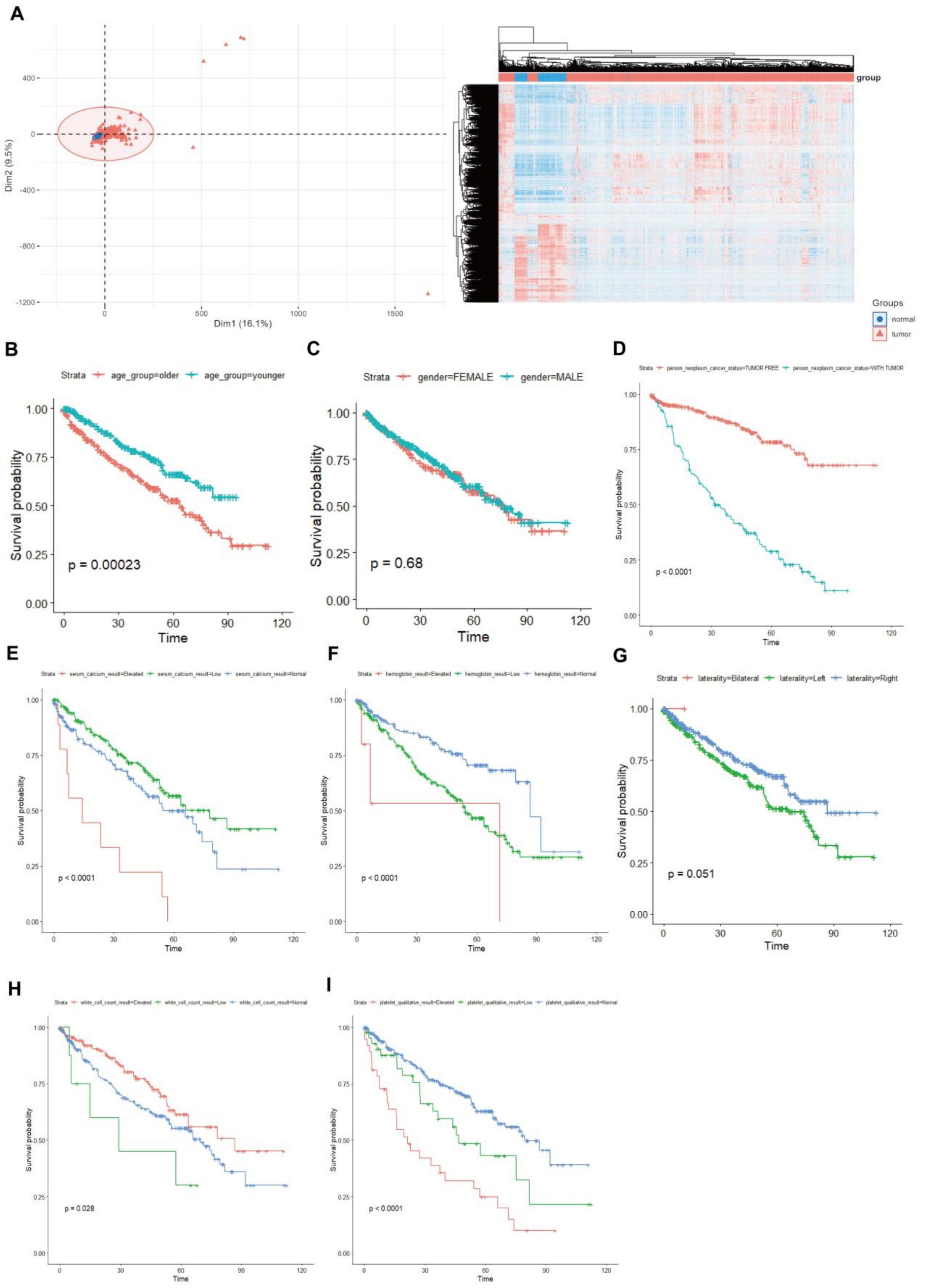

Figure S1. (A) Principal component analysis and heatmap showing the global transcriptomic differences between KIRC and normal kidney tissues. (B-I) Kaplan–Meier survival analyses of KIRC patients stratified by age, sex, laterality, hemoglobin level, platelet count, serum calcium level, tumor status and white blood cell count.

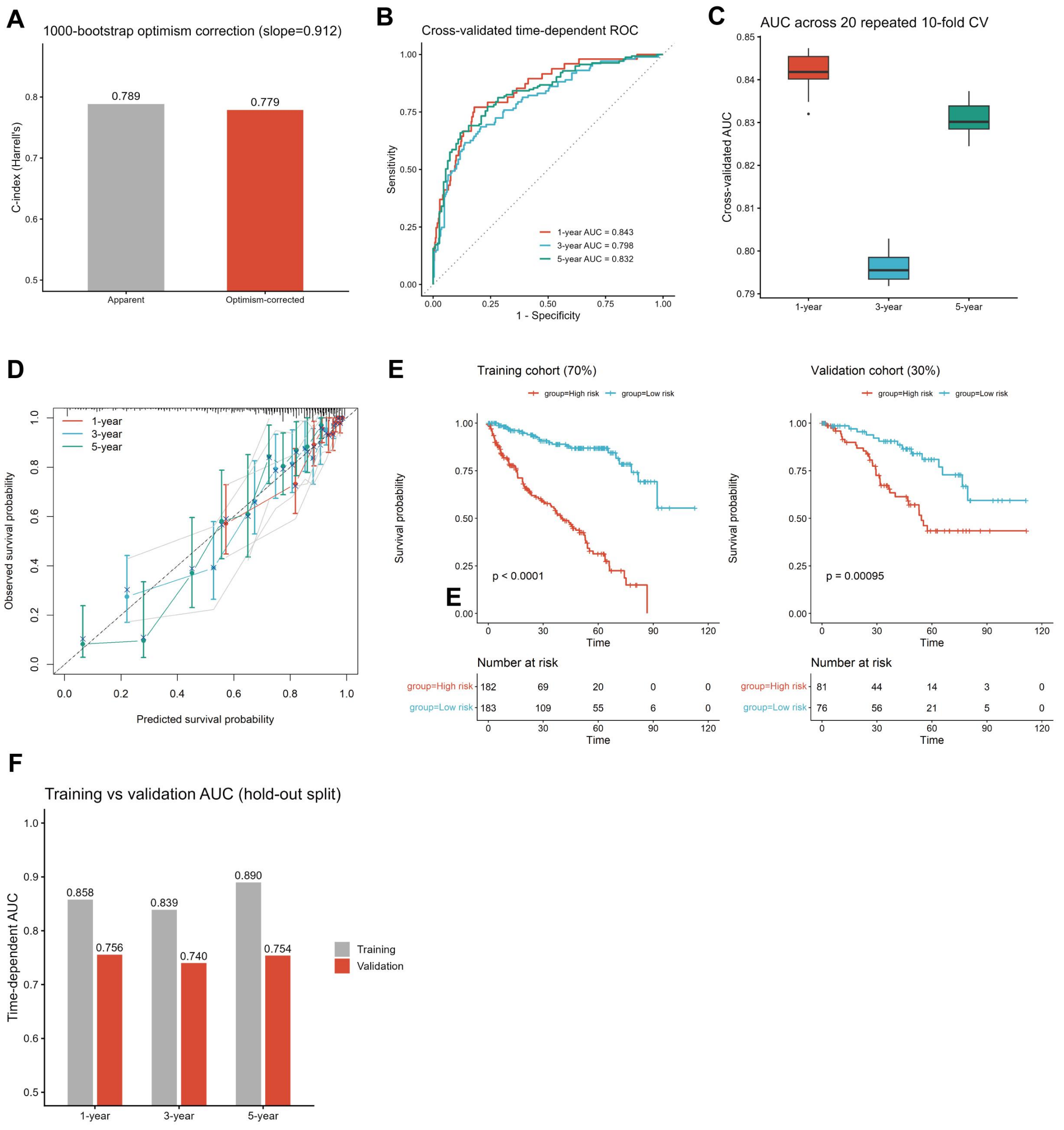

Figure S2. (A) Bootstrap-based optimism correction of Harrell's C-index. (B) Cross-validated time-dependent ROC curves at 1, 3, and 5 years. (C) Distribution of AUCs across repeated 10-fold cross-validation. (D) Calibration curves for predicted versus observed survival probabilities. (E) Kaplan–Meier survival curves for the high- and low-risk groups in the training and hold-out validation cohorts. (F) Comparison of time-dependent AUCs between the training and validation cohorts.

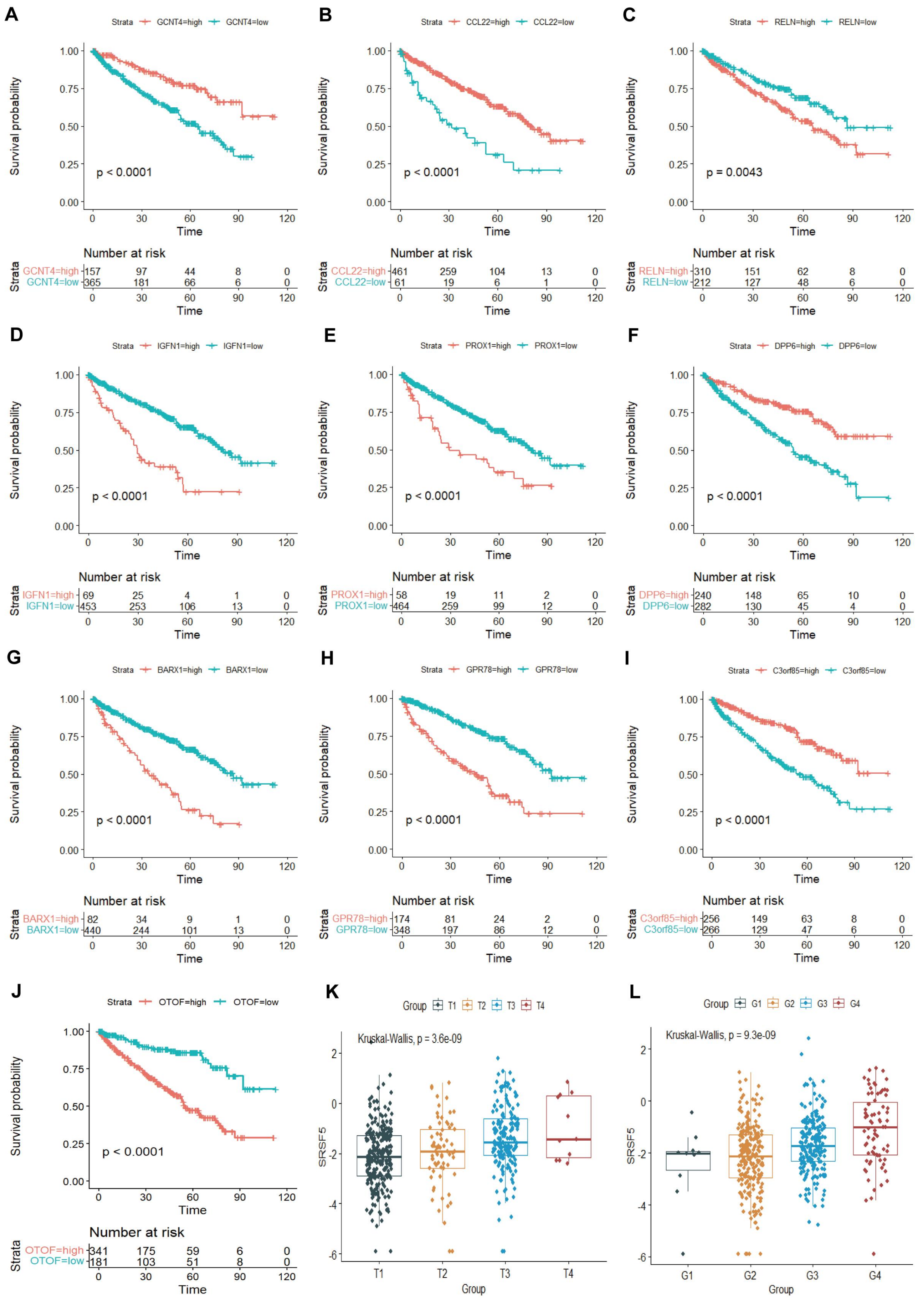

Figure S3. (A–J) Kaplan–Meier overall survival curves stratified by high and low expression of GCNT4, CCL22, RELN, IGFN1, PROX1, DPP6, BARX1, GPR78, C3orf85, and OTOF, respectively. (K, L) Distribution of OTOF expression across pathological T stages and histological grades, respectively.

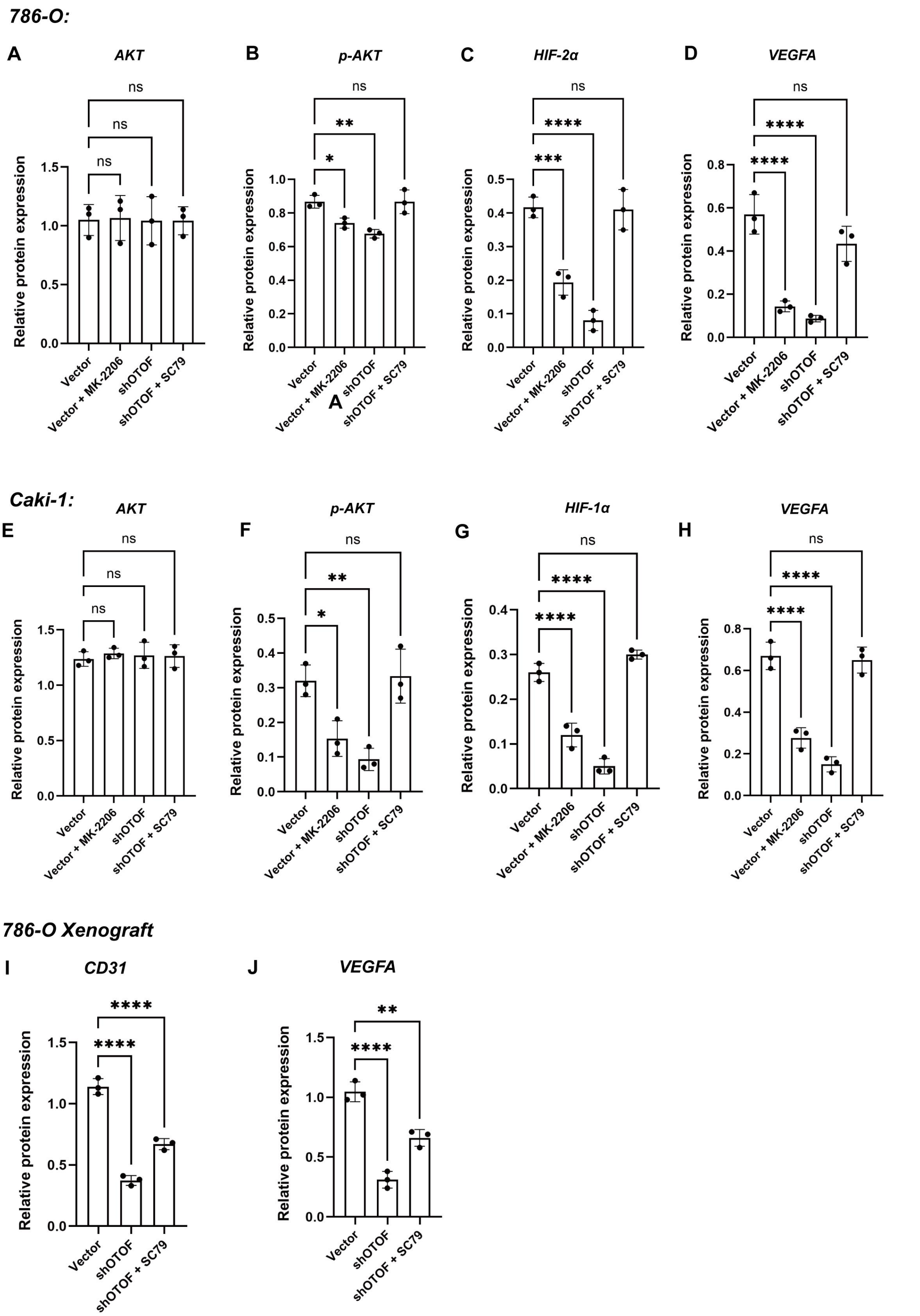

Figure S4. (A–D) Western blot quantification of AKT, p-AKT, HIF-2α, and VEGFA in 786-O cells. (E–H) Western blot quantification of AKT, p-AKT, HIF-1α, and VEGFA in Caki-1 cells. (I–J) Western blot quantification of CD31, and VEGFA in 786-O xenograft tumors.

Supplementary Table S1. The key characteristics of all ten signature genes.

Gene	OTOF	GPR78	C3orf85	BARX1	DPP6	IGFN1	GCNT4	PROX1	CCL22	RELN
LASSO coefficient	0.084479	0.068408	−0.016206	0.0136923	−0.037706	0.011132	−0.028617	0.0348226	−0.010519	0.0108836
Hazard ratio	1.25	1.27	0.92	1.09	0.85	1.07	0.81	1.16	0.85	1.09
P value of survival association	<0.0001	<0.0001	<0.0001	<0.0001	<0.0001	<0.0001	<0.0001	<0.0001	<0.0001	0.0043
Differential Expression Adjusted P value	7.183E−45	5.039E−05	8.23E−31	2.477E−20	1.18E−30	2.255E−26	5.03E−24	1.507E−91	9.463E−10	3.987E−05
Previous ccRCC evidence	yes	no	no	yes	yes	yes	yes	yes	yes	no

Supplementary Table S2. Internal validation results.

Metric	Value
C-index (apparent)	0.789
C-index (optimism-corrected)	0.779
Calibration slope (corrected)	0.912
CV AUC 1-year	0.842
CV AUC 3-year	0.796
CV AUC 5-year	0.831
Hold-out validation AUC 1-year	0.756
Hold-out validation AUC 3-year	0.74
Hold-out validation AUC 5-year	0.754
